# Antithrombotic Therapy in the Elderly with Cardiovascular Disease: Walking the Tightrope Between Efficacy and Bleeding Risk—A Narrative Review

**DOI:** 10.3390/jcm14207340

**Published:** 2025-10-17

**Authors:** Alessandro Sciahbasi, Simona Minardi, Nicolò Salvi, Fabio Infusino, Antonino Granatelli

**Affiliations:** 1 Interventional Cardiology, Sandro Pertini Hospital—ASL RM2, 00157 Rome, Italy; nicolo.salvi@aslroma2.it (N.S.); fabio.infusino@aslroma2.it (F.I.); antonino.granatelli@aslroma2.it (A.G.); 2 Cardiology, Sant’Eugenio Hospital—ASL RM2, 00144 Rome, Italy; simona.minardi@aslroma2.it

**Keywords:** elderly, antithrombotic therapy, dual antiplatelet therapy, anticoagulants, DOACs, frailty, atrial fibrillation, PCI, factor XIa inhibitors

## Abstract

The number of elderly patients requiring antithrombotic therapy (for example, those with atrial fibrillation, ischemic heart disease, peripheral arterial disease, or venous thrombo-embolism) is increasing worldwide due to population aging. These patients are often frail and therefore at increased risk of both thromboembolic events and bleeding complications during antithrombotic treatment. Therapeutic decision-making is further complicated by the underrepresentation of older adults in large randomized trials and the resulting scarcity of age-specific, evidence-based data. As a result, their management is not guided by specific recommendations but rather relies on clinician evaluation in an individualized, patient-by-patient approach. The aim of this narrative review is to discuss the current optimal therapeutic strategies for the management of elderly patients in different clinical conditions requiring antithrombotic therapy. We analyze the efficacy and safety of the different anti-thrombotic drugs and guidelines indications by discussing the clinical data available from randomized as well as observational studies. At the same time, we focus into the future of antithrombotic therapy presenting new drugs and new strategies for the management of elderly patients requiring antithrombotic therapy.

## 1. Introduction

With the global population aging, the absolute number of elderly patients requiring antithrombotic therapy (such as patients with atrial fibrillation, ischemic heart disease, peripheral venous and arterial disease, etc.) is also increasing, representing a group particularly vulnerable to thromboembolic events and bleeding complications. It has been estimated that more than 70% of adults over the age of 70 years develop atherosclerotic cardiovascular disease (ASCVD) [1], and the prevalence of atrial fibrillation is 10–17% in patients aged ≥80 years [2], including conditions requiring intensive antiplatelet and/or anticoagulant therapy.

The complexity of the management of antithrombotic therapy in the elderly arises from the age-related alterations in homeostasis observed in the elderly, along with vascular frailty, multiples comorbidities, and changes in drug absorption, distribution, metabolism, and clearance, which make the antithrombotic treatment of older patients particularly challenging, with higher rates of adverse effects from therapies. The therapeutic challenge is further complicated by the fact that elderly patients are generally excluded from large randomized trials, and evidence-based clinical data are limited. As a result, their management is not guided by precise recommendations but rather relies on physician judgment and an individualized, patient-by-patient approach.

This review aims to provide a comprehensive overview of the optimal therapeutic strategies for the management of elderly patients in different clinical conditions requiring antithrombotic therapy, focusing on the efficacy and safety of the different drugs available. By synthesizing current evidence, this review will also give a glimpse into the future of antithrombotic therapy.

## 2. Methodology

All studies that reported antithrombotic administration in the elderly with cardiovascular disease were evaluated. PubMed, EMBASE, and Cochrane Central Register and reference lists of relevant studies including “elderly”, “antithrombotic”, “anticoagulant”, “antiplatelet” were searched up until June 2025. The search strategy used was (Elderly) AND (Antithrombotics OR Anticoagulant OR Antiplatelet) OR (Elderly) AND (Aspirin OR Clopidogrel OR Warfarin OR Dabigatran OR Rivaroxaban OR Apixaban OR Edoxaban OR Factor XI inhibitors). No restriction was placed on study settings nor geographic areas, and only fully published papers were considered.

## 3. Definition of “Elderly” Patient

There is currently no universally accepted definition of an “elderly patient”: The World Health Organization (WHO) defines elderly individuals as those over 60 years of age in industrialized countries [3]. Differently, the National Institutes of Health (NIH) defines elderly people as those older than 65 and further classifies them into “young-old” (65–74), “middle-old” (75–84), and “oldest-old” (≥85) [4]. Moreover, many practice guidelines do not adequately define “elderly patients” [5], defining a “cutoff” of age. In this review, according to many studies, we decided to focus on individuals aged ≥75 years as representative of this group of patients.

## 4. Age-Related Changes in the Thrombotic Pathway

In elderly individuals, multiple alterations of the hemostatic, coagulative, and fibrinolytic systems have been observed [6]. In particular, platelet function is significantly affected by aging: platelet count tends to decrease slightly with age, but platelet reactivity increases [7,8]. Adenosine diphosphate (ADP)-induced platelet aggregation, as well as other platelet agonist mediated aggregation [9], becomes more pronounced with age, and the concentration of ADP required to induce aggregation decreases with advancing age [10]. All these modifications contribute to increasing the thrombotic risk (Table 1).

Aging is associated with reduced inhibitory mechanisms of platelet activation, as proven by the decrease in the number in platelet receptors for prostaglandin I2 (PGI2) and the circulating levels of PGI2 itself [11]. Another potent inhibitor of platelet activation, nitric oxide (NO), is significantly reduced in the elderly due to the typical age-related endothelial dysfunction and to the increase in intraplatelet production of reactive oxygen species deputies to the oxidative degradation of NO [12,13].

The coagulation cascade is, at the same time, altered in the elderly, typically exhibiting a procoagulant state, as evidenced by the increased D-dimer levels. A well-documented rise in procoagulant factors (factors VII, VIII, IX, X, XII, von Willebrand factor, prekallikrein, and high-molecular-weight kininogen) along with a chronic low-grade inflammatory state is often observed in older adults [14], exacerbating thrombotic risk and worsening outcomes in events such as acute coronary syndromes (ACS).

Finally, aging affects the fibrinolytic system: the expression of plasminogen activator inhibitor-1 (PAI-1), a major antifibrinolytic factor, increases with age, thereby further tipping the hemostatic balance towards thrombosis [14].

## 5. Increased Bleeding Risk in Elderly

Many factors that increase ischemic risk in elderly patients, such as age-related physiological changes and common comorbidities, also predispose them to bleeding [15] (Table 1). Advancing age is associated with structural and functional changes in the vasculature, including loss of soft-tissue support for the cutaneous microvasculature, compounded by increased capillary pressure [16]. Moreover, age affects the pharmacokinetics and pharmacodynamics of multiple drugs because hepatic and renal function decline progressively, impairing drug metabolism and clearance [17]. Lower serum albumin levels alter drug binding and distribution, while increased sensitivity to other molecules, such as antithrombotics, can lead to excessive anticoagulation and unstable INR levels [17,18]. Advancing age is associated with increased risk of polypharmacy and related drug interactions [19]: the concomitant use of medications such as Non Steroidal Anti Inflammatory Drugs (NSAIDs), along with alcohol consumption, significantly increases the risk of gastrointestinal and intracranial bleeding. These agents act synergistically with antithrombotic drugs to impair mucosal integrity and platelet function [20].

Anemia, highly prevalent in the elderly, is another contributor to bleeding risk. It may result from chronic diseases, nutritional deficiencies, or occult bleeding [21]. Importantly, anemia itself impairs platelet function and reduces the availability of ADP, compromising the hemostatic response [22].

Hypertension, present in the majority of older adults, significantly increases the risk of intracerebral bleeding, especially when systolic pressure exceeds 160 mmHg [23]. Hypertensive vasculopathy leads to microvascular damage and the development of cerebral microbleeds, further exacerbated by antithrombotic therapy [24].

Finally, elderly individuals are more prone to falls, a consequence of reduced balance, vision, proprioception, and cognitive function [25]. Falls represent a major clinical concern, especially due to the potential for traumatic intracranial hemorrhage in patients on antithrombotic therapy.

Globally, the increased bleeding risk of elderly patients highlights the clinical medical dilemma between reducing antithrombotic doses and the risk of under-treatment exposing patients to a higher thrombotic risk. Consequently, physicians should carefully balance the risk -benefit of a less intense antithrombotic therapy.

## 6. Atherosclerotic Cardiovascular Disease

Age is a significant risk factor for ASCVD, particularly coronary artery disease (CAD), that is highly prevalent among the elderly. The main treatment for CAD is antiplatelet therapy, especially for secondary prevention after a percutaneous coronary intervention (PCI) [26]. The most used oral antiplatelet agents are aspirin, which is an irreversible inhibitor of the platelet cyclooxygenase-1, and P2Y12 receptor inhibitors, such as Clopidogrel, Prasugrel, and Ticagrelor. The use of intravenous antiplatelet agents, such as GPIIb/IIIa inhibitors and Cangrelor, is limited to specific clinical situations, with particular caution required in elderly patients. Main studies on patients with atherosclerotic cardiovascular disease are presented in Table 2.

### 6.1. Primary Prevention

The benefit of antithrombotic therapy for primary prevention remains controversial. Recent trials have shown little to no benefit in patients without established ASCVD, along with a consistent increase in bleeding risk [27,28]. Some of these studies randomized elderly patients to receive aspirin 100 mg daily or placebo and found no significant difference in the primary endpoint of cardiovascular death, stroke, or myocardial infarction, while observing a significant increase in the risk of major bleeding [29,30]. No other endpoints, such as dementia or persistent physical disability, were found to be reduced by aspirin intake [31].

**Figure 1:**
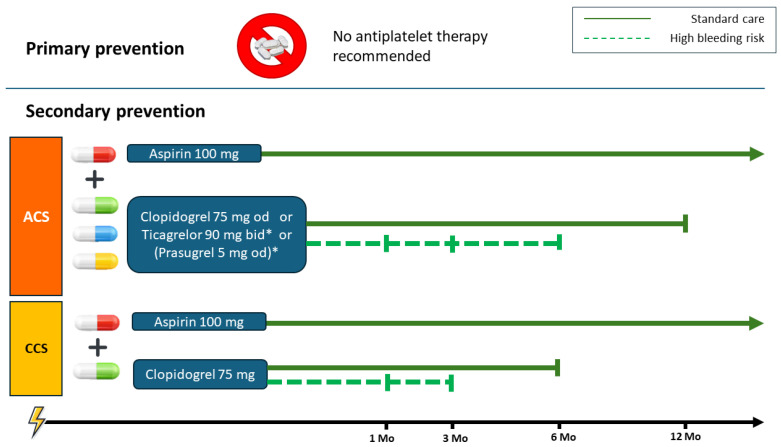
Antithrombotic therapy in elderly patients undergoing PCI in acute and chronic settings. In patients with ACS, guidelines recommend 12 months of DAPT, followed by lifelong SAPT (Aspirin or Clopidogrel). The P2Y12 inhibitor can be either clopidogrel or ticagrelor. The use of prasugrel in patients over 75 years is debated: some guidelines recommend a reduced dose of 5 mg once daily, as well as in patients weighing <60 kg. * A history of ischemic or hemorrhagic stroke or TIA is a contraindication to prasugrel, while a history of hemorrhagic stroke is a contraindication to ticagrelor. Shorter DAPT durations (e.g., 6 or 3 months) are recommended in patients at high bleeding risk. In patients with CCS, guidelines recommend 6 months of DAPT (aspirin + clopidogrel), followed by lifelong SAPT (aspirin or clopidogrel). In high bleeding risk patients, shorter DAPT durations (e.g., 3 or 1 month) are advised. Age-based analyses from RCTs indicate that extending DAPT over 12 months in ACS or over 6 months in CCS provides no additional ischemic protection in elderly patients but significantly increases bleeding risk. ACS: Acute Coronary Syndrome, CCS: Chronic Coronary Syndrome, DAPT: Dual Antiplatelet Therapy, RCT: Randomized Controlled Trial, SAPT: Single Antiplatelet Therapy, TIA: transient ischemic attack.

Based on these findings, American guidelines do not recommend the routine use of Aspirin for primary prevention in patients over 70 years of age [32] (Figure 1), whereas there are no specific recommendations for the elderly in the European guidelines.

### 6.2. Secondary Prevention

Single antiplatelet therapy (SAPT) is the cornerstone of long-term secondary prevention of CAD in the general population as well as in elderly patients [33]. Although most available studies are outdated and included only a small proportion of patients over 75 years of age, current recommendations for patients with a history of CAD without an indication for oral anticoagulant therapy (OAT) (see below) or absolute contraindications to antithrombotic therapy support lifelong SAPT with either Aspirin or Clopidogrel, regardless of age [34].

The gold standard antithrombotic strategy in the period following an ACS, or, regardless of clinical presentation, after a PCI, is dual antiplatelet therapy (DAPT). This consists of Aspirin in combination with a P2Y12 receptor inhibitor (Clopidogrel, Prasugrel or Ticagrelor) [34,35], which inhibits platelet aggregation by blocking the ADP binding site.

GP IIb/IIIa inhibitors have been extensively studied in the era preceding the routine use of DAPT [36,37]. Today, there is no strong evidence supporting their routine use in patients undergoing coronary angiography. However, they may be considered as a bailout option in cases of no-reflow or thrombotic complications during PCI [35]. Another potential indication for GP IIb/IIIa inhibitors is in patients requiring urgent non-cardiac surgery shortly after the index event or PCI with stent implantation, when interruption of DAPT would pose a high risk [38]. There are no absolute contraindications to the use of GP IIb/IIIa inhibitors in elderly patients, but due to the increased bleeding risk in this population, these agents should be used with extreme caution.

Cangrelor is an intravenous, direct, reversible, short-acting P2Y12 receptor inhibitor. Several randomized clinical trials (CHAMPION PCI, PLATFORM, and PHOENIX) [39,40,41] have evaluated its use compared to Clopidogrel during PCI in acute coronary syndromes. The results of these studies included in a meta-analysis showed that Cangrelor reduces ischemic events at the cost of an increased risk of minor bleeding [42]. Evidence on its use in combination with Ticagrelor or Prasugrel is limited. Today, Cangrelor may be considered in P2Y12 inhibitor–naïve ACS patients, especially when oral therapy is not feasible, such as in emergent PCI, cardiogenic shock, or mechanically ventilated patients. Another potential indication for Cangrelor is the treatment of patients requiring urgent non-cardiac surgery shortly after an ACS or PCI with stent implantation, when interruption of DAPT would pose a high risk [38]. Recently, an Italian study based on a post hoc analysis of the multicenter ARCANGELO study evaluated the safety and efficacy of Cangrelor in older patients (≥75 years) undergoing PCI for ACS (995 patients globally, 215 aged ≥75) [43]. The results showed that although Major Adverse Cardiac Events (MACE) were more frequent in the elderly (3.3% vs. 0.9%, *p* = 0.017), there were no significant differences in overall bleeding rates (Bleeding Academic Research Consortium—BARC criteria) comparing patients ≥75 and <75 years (7.4% vs. 4.6%, *p* = 0.1179). Most bleeding events were mild (BARC 1–2) and occurred within 48 h after PCI, while moderate-to-severe bleeding (BARC 3–5) was very low in both groups (0.9% in elderly, 0.4% in younger). Treatment-emergent adverse events were higher in the elderly (36.3% vs. 19%, *p* < 0.0001), but events directly correlated to Cangrelor use were rare and similar between groups (0.9% vs. 0.5%, respectively) [43].

## 7. Dual Antiplatelet Therapy in Acute Coronary Syndrome in Elderly Patients

The superiority of DAPT in patients with ACS has been recognized since 2001, when the CURE study (a randomized trial involving 12,562 patients with non–ST-elevation myocardial infarction-NSTEMI) compared the efficacy of DAPT with Aspirin and Clopidogrel versus Aspirin monotherapy. The composite outcome of cardiovascular death, myocardial infarction (MI), stroke, or severe ischemia was significantly reduced in the group receiving combination therapy, although this benefit came at the cost of a modest increase in non-fatal bleeding events. As a result, clopidogrel in combination with Aspirin became the cornerstone of DAPT, supported by numerous subsequent trials conducted in high-risk populations, with no significant safety concerns in elderly patients, making it a widely used agent in this group [44].

Aspirin and clopidogrel remained the standard dual antiplatelet therapy until 2007, when the TRITON-TIMI 38 trial [45] and, a year later, the PLATO trial [46] were published, evaluating, respectively, the efficacy of Aspirin plus Prasugrel and Aspirin plus Ticagrelor, in the context of ACS.

The TRITON-TIMI 38 trial demonstrated a significant reduction in the primary endpoint of death from cardiovascular causes, non-fatal MI or non-fatal stroke in the Aspirin + Prasugrel group compared to the Aspirin + Clopidogrel group (9.9% vs. 12.1%, respectively, *p* < 0.001), accompanied by a significant increase in major bleeding events in the Prasugrel group. Such excess in bleeding was particularly high in elderly patients, resulting in a neutral net clinical benefit [45]. The increased risk of bleeding among elderly patients has been related to an increased exposure to the active metabolite of the drug, which was greater with advanced age [47]. Because of these findings, a full dose of Prasugrel is generally not recommended in patients ≥75 years of age, even though the U.S. Food and Drug Administration still considers the use of Prasugrel 10 mg for patients older than 75 years at high ischemic risk in the absence of contraindications (prior cerebrovascular event or active bleeding). A therapeutic option in clinical practice is to reduce the maintenance dose among elderly patients to prasugrel 5 mg [48]. Prasugrel 5 mg was compared to Clopidogrel in the TRILOGY ACS (Targeted Platelet Inhibition to Clarify the Optimal Strategy to Medically Manage Acute Coronary Syndromes) study [49] and in the ELDERLY ACS II (Elderly Acute Coronary Syndrome 2) trial [50]. In these studies, Prasugrel 5 mg provided more potent platelet inhibition compared to Clopidogrel among elderly patients, but the difference was small and did not translate into relevant clinical benefits.

In the PLATO trial, patients treated with the combination of Aspirin and Ticagrelor showed a primary endpoint rate of death from cardiovascular causes, non-fatal MI or non-fatal stroke of 9.8%, compared to 11.7% in patients treated with Aspirin + Clopidogrel (*p* < 0.001), with no significant difference in major bleeding between the two groups (*p* = 0.43) [46]. The difference in bleeding was not significant across age subgroups, so that Ticagrelor 90 mg twice daily is recommended after ACS, with no specific age-related recommendations [51]. Other randomized and observational studies comparing Ticagrelor and Clopidogrel in combination with Aspirin in elderly patients with ACS have yielded conflicting results. In the Bremen STEMI Registry, Ticagrelor was associated with a reduction in ischemic events without a significant increase in bleeding [52]. In contrast, the SWEDEHEART registry (Swedish Web System for Enhancement and Development of Evidence-Based Care in Heart Disease Evaluated According to Recommended Therapies) showed that Ticagrelor had comparable efficacy to Clopidogrel, but it was associated with higher rates of bleeding and mortality [53]. The POPular AGE (Ticagrelor or Prasugrel Versus Clopidogrel in Elderly Patients With an Acute Coronary Syndrome and a High Bleeding Risk: Optimization of Antiplatelet Treatment in High-Risk Elderly) trial randomized 1:1 patients aged 70 years or older with NSTE-ACS to receive clopidogrel or a new P2Y12 inhibitor (ticagrelor or prasugrel) [54]. Since patients treated with ticagrelor were 95% of the new P2Y12 inhibitors group, the results can be interpreted as a comparison between Clopidogrel and Ticagrelor. Clopidogrel led to fewer bleeding events without an increase in the combined endpoint of all-cause death, myocardial infarction, stroke, and bleeding, so it could be an alternative P2Y12 inhibitor, especially for elderly patients with a higher bleeding risk [54].

In summary, all these studies highlight the clinical challenge of achieving a balance between ischemic and bleeding risk in elderly patients, and a tailored therapy for the single patient should be advisable in order to obtain the best balance.

Take-home message: The newer P2Y12 inhibitors provide more effective protection against ischemic events and are considered the first-line option in combination with aspirin after an ACS, even in older patients. However, due to their association with a higher risk of bleeding, these agents should be used with caution in elderly individuals with a high bleeding risk. A reduced dose of prasugrel (5 mg) should be considered in patients aged ≥75 years. Clopidogrel, in combination with aspirin, remains a valid alternative for elderly patients at high bleeding risk.

### DAPTDuration After an Acute Coronary Syndromes

DAPT duration in ACS is generally recommended for 12 months, especially following PCI with stent implantation. However, to reduce bleeding risk, the duration may be shortened to 6 months or up to 1–3 months based on the patient’s risk profile and the type of treatment performed. The age is not “per se” a determinant criterion to reduce the DAPT duration: the real determinant is the individual risk of bleeding. The PRECISE DAPT score [55] is a five-items score including age (creatinine clearance, hemoglobin, white blood cell count, and previous spontaneous bleeding) aimed to identify patients (score ≥ 25) in whom DAPT should be shortened. Another important factor when considering DAPT duration in the elderly is the evaluation of geriatric status, with particular attention to the physical frailty that has been demonstrated as a predictor of poor outcomes in patients with ACS [56]. Frailty assessment should be considered as a determinant item when evaluating DAPT in older adults with ACS.

In patients with ACS who have tolerated DAPT for 12 months without bleeding events and who present with a low bleeding risk and high thrombotic risk, the treatment may be extended beyond 12 months. This treatment option is supported by the results of the PEGASUS-TIMI 54 trial [57], which enrolled 21,162 patients with a history of MI and at least one high-risk feature such as age ≥ 65 years, diabetes mellitus, recurrent MI, multivessel coronary artery disease, or chronic kidney disease. Patients were randomized to receive, in addition to aspirin, either ticagrelor 90 mg twice daily, ticagrelor 60 mg twice daily, or placebo. The trial demonstrated that extending DAPT beyond 12 months with ticagrelor (especially at 60 mg twice daily) reduced the risk of cardiovascular events with a modest increase in major bleeding. As a result, Ticagrelor 60 mg twice daily is the preferred agent for long-term DAPT, while Clopidogrel remains an alternative when Ticagrelor is not suitable. However, age specific sub-analysis of other studies suggest that elderly patients are not ideal candidates to achieve the optimal risk-benefit balance with extended DAPT. An example is the analysis of the PRODIGY trial [58] that compared the efficacy and safety of 24-month versus 6-month DAPT in elderly (≥75 years) versus non-elderly (<75 years) patients. The study showed that prolonged DAPT did not significantly reduce ischemic events in the elderly with a significant increase in bleeding events. According to this evidence, clinical decisions in elderly patients should be carefully evaluated when considering extended DAPT duration.

## 8. Dual Antiplatelet Therapy in Chronic Coronary Syndrome in Elderly Patients

In patients with CCS, DAPT with Aspirin + Clopidogrel is the recommended treatment strategy after PCI, especially following stent implantation [34]. There is no indication for other P2Y12 inhibitors in the context of CCS and currently, guidelines recommend 6 months of DAPT in CCS even though shorter durations might be considered in specific situations [34]. Compared to 6-month DAPT, longer durations such as 12 or 24 months are non-inferior in preventing ischemic events while increasing bleeding risk [59,60]. Recently, different studies evaluated the possibility of a short (1 to 3 months) dual-antiplatelet therapy in CCS, showing good antithrombotic safety and a reduced bleeding risk. In particular, in a post hoc analysis of the TWILIGHT trial that included CCS patients undergoing PCI, ticagrelor monotherapy after 3 months of DAPT appeared to be safe and was not associated with increased risks of ischemic events irrespective of sex [61]. Moreover, a recent analysis from threeprospective international studies (XIENCE Short DAPT Program) showed that in patients undergoing PCI with everolimus-eluting stents, 1- and 3-month DAPTs were associated with similar risk for ischemic events, but the 1-month DAPT strategy resulted in less clinically relevant bleeding [62].

Specific and dedicated studies in elderly patients with CCS undergoing PCI are lacking, even though a recent analysis performed using data from 10,487 elderly patients across 5 Korean randomized trials showed that standard DAPT strategy may increase bleeding risk without reducing ischemic events [63].

Similarly to ACS patients, in order to reduce the risk of bleeding, the use of scores such as the PRECISE-DAPT or indicators of geriatric status are of great value to identify patients requiring a shortening of DAPT duration (e.g., 3 or 1 month) [55,64].

In summary, current guidelines on DAPT [65] do not provide specific recommendations for elderly CCS patients. The standard 6-month DAPT could be reduced to 1 or 3 months in elderly patients after assessing bleeding risk and geriatric status, whereas longer durations are not advisable.

## 9. Anticoagulant Therapy in Elderly

The indications for anticoagulant therapy are numerous, but the most common include the prevention of cardioembolic stroke in patients with atrial fibrillation, the prevention and treatment of pulmonary embolism and deep vein thrombosis, and the period following heart valve replacement. Other possible indications include left ventricular thrombosis, the post-ablation period for ventricular tachycardia, and the treatment of disseminated intravascular coagulation.

A significant percentage of individuals aged 75 and older are prescribed oral anticoagulants, primarily for AF and venous thromboembolism (VTE), which together account for nearly 90% of all indications [66]. The prevalence of these two conditions increases with age: AF due to age-related structural changes in the atria and comorbidities, and VTE due to endothelial dysfunction, venous stasis, and heightened coagulability typical of the elderly. Around 85% of AF patients and two-thirds of those with VTE are aged 65 or above, and their clinical outcomes are typically worse than in younger populations [67,68].

Among oral anticoagulants, vitamin K antagonists (VKAs) were the first to be developed. They act by inhibiting the enzyme vitamin K epoxide reductase, thereby preventing the activation of vitamin K-dependent coagulation factors (proteins C and S and factors II, VII, IX, and X).

Over the past decade, direct oral anticoagulants (DOACs) have been introduced into clinical practice to overcome some of the limitations of the VKAs (variable anticoagulant effect, slow onset of action). These drugs selectively inhibit either factor Xa (e.g., Rivaroxaban, Apixaban, Edoxaban) or thrombin (Dabigatran). DOACs are characterized by good oral bioavailability, rapid onset of action, and predictable pharmacokinetics and are suitable for a broad range of patients (Table 2).

Apixaban is generally considered the most suitable DOAC for elderly patients due to its favorable bleeding profile, particularly with respect to gastrointestinal (GI) bleeding and intracranial hemorrhage (ICH). A dose reduction to 2.5 mg twice daily is required when patients are aged ≥80 years and have at least one additional criterion (body weight ≤ 60 kg or serum creatinine ≥ 1.5 mg/dL). Apixaban is also well tolerated in frail patients and in those with high fall risk, while its twice-daily schedule may pose adherence challenges in those with cognitive impairment.

Edoxaban represents a valid alternative, especially because of its once-daily regimen, which may improve adherence in cognitively impaired elderly. The dose should be reduced to 30 mg once daily in patients with low body weight (≤60 kg), moderate renal impairment (creatinine clearance 15–50 mL/min), or when combined with P-glycoprotein inhibitors. It is also considered reasonable in patients at high fall risk and in those with a history of GI disease, particularly at the reduced 30 mg dose.

Rivaroxaban requires a dose reduction to 15 mg once daily in patients with moderate renal impairment (creatinine clearance 15–49 mL/min). It does not mandate specific age- or weight-based adjustments, but caution is advised in patients ≥75 years due to the increased risk of GI bleeding. While its once-daily regimen may favor adherence, rivaroxaban is less suitable in elderly patients with high fall risk or significant GI pathology, given its higher bleeding risk compared with apixaban and edoxaban.

Dabigatran should be used with greater caution in the elderly because of its renal dependence and higher risk of GI bleeding. Although no formal dose reduction criterion is mandated, many guidelines recommend considering the 110 mg twice daily dose in patients aged ≥80 years or in those with increased bleeding risk. It is less suitable in frail or cognitively impaired elderly, as its twice-daily regimen may hinder adherence, and it is also less favored in fall-prone patients due to its higher ICH risk.

Clinical data have shown non-inferiority, and in some cases, superiority of DOACs compared to VKAs in preventing cardioembolic events in non-valvular AF and treating and preventing VTE, while VKAs are still considered the treatment of choice for specific indications, including mechanical heart valves, moderate/severe mitral stenosis, end-stage renal failure, and the antiphospholipid syndrome [69,70].

Apixaban is a direct factor Xa inhibitor, taken twice daily. It has a relatively low degree of renal elimination (~25%) and is metabolized primarily via CYP3A4 and P-glycoprotein (P-gp) (~75%) [71]. Apixaban is a hydrophilic drug, so a muscle mass reduction is associated with a lower volume distribution and higher plasma concentration. It is estimated that a body weight below <60 kg results in up to 27% higher Apixaban plasma levels [72]. A dose reduction is recommended if two or more of the following criteria are present: age ≥ 80 years, body weight ≤ 60 kg, or serum creatinine ≥ 1.5 mg/dL (133 μmol/L) [71]. Among all DOACs, Apixaban has consistently shown the most favorable safety profile in older adults, particularly regarding the risk of major bleeding and intracranial hemorrhage (ICH) [73]. It is well tolerated in frail, multimorbid, and cognitively impaired individuals and has a lower likelihood of significant drug–drug interactions [74,75]. These properties make apixaban a preferred choice in many older patients, especially those with high bleeding risk or polypharmacy.

Edoxaban is also a direct factor Xa inhibitor, taken once daily. It is eliminated approximately 50% by the kidney and 50% by CYP3A4 [71]. As well as the other DOACs, it is a hydrophilic drug, so that a body weight below <60 kg results in up to 40% higher Edoxaban plasma levels [72]. A dose reduction is recommended if one or more of the following criteria are present: Creatinine Clearance (CrCl) between 15 and 50 mL/min, body weight ≤ 60 kg, or concurrent use of potent P-gp inhibitors. Edoxaban has demonstrated good efficacy and safety in older adults, with a risk-benefit profile comparable to Apixaban, particularly in patients with moderate renal impairment or frailty [76,77]. It may be a suitable option in patients who require a simplified, once-daily regimen.

Rivaroxaban is another factor Xa inhibitor, taken once daily with food to enhance absorption. It is cleared renally (~33%) and metabolized via CYP3A4 and P-gp. A dose reduction is recommended in patients with a CrCl between 15 and 50 mL/min [71]. In older adults, Rivaroxaban has been associated with a higher risk of gastrointestinal bleeding compared to other DOACs, in particular compared to Apixaban [73,74,75]. As such, it may not be the optimal choice for very elderly, frail patients or those with a history of gastrointestinal pathology. The American Geriatrics Society has advised caution or avoidance of Rivaroxaban in patients aged ≥75 years unless compelling reasons exist [78].

Dabigatran is the only DOAC that directly inhibits thrombin (factor IIa), taken twice daily. It has a high degree of renal elimination (>80%) and is not metabolized by the CYP system, though it is a substrate of P-gp. A dose reduction is recommended for patients aged ≥80 years, or earlier in those with impaired renal function (CrCl 30–50 mL/min) or increased bleeding risk [71]. Due to its dependence on renal clearance, Dabigatran requires close monitoring of kidney function in older adults, especially those over 75 years. It has been associated with a higher risk of gastrointestinal bleeding than Apixaban and Edoxaban and may accumulate in patients with age-related renal decline [73,74,75]. Because of these concerns, its use in older, frail, or cognitively impaired individuals is generally less preferred unless there are compelling indications [78].

Despite the advantages of DOACs over VKAs, recently the FRAIL-AF trial reported a higher incidence of bleeding events without benefit in terms of switching from VKAs to DOACs in frail older patients, suggesting that elderly people that have been long-term treated with VKAs should continue this therapy, especially when INR is >70% time in therapeutic range and no adverse effects are reported [79].

## 10. New Anticoagulants Under Development

In recent years, the limitations of existing antithrombotic therapies, most notably their elevated risk of bleeding, have driven the search for new agents capable of preventing thrombosis while causing minimal disruption of normal hemostasis. Insights gained from genetic disorders and various experimental animal models have drawn increasing attention to the contact phase of the intrinsic coagulation pathway as a promising target [80].

One particularly important observation is that individuals with congenital factor XI (FXI) deficiency tend to have a significantly lower incidence of thrombotic events, yet they experience only a mild tendency to bleed. Conversely, elevated plasma levels of FXI have been associated with an increased risk of both initial and recurrent VTE. These findings have positioned FXI and its active form, FXIa as attractive therapeutic targets for the prevention of cardiovascular and thromboembolic events [80,81,82]

Several drug classes targeting FXI/FXIa have been developed: antisense oligonucleotides (such as IONIS-FXIRx and Fesomersen), which suppress hepatic production of FXI; monoclonal antibodies (including Osocimab, Abelacimab, Xisomab 3G3, and MK-2060), which bind to FXI or FXIa to block activation; and small molecule inhibitors (such as Milvexian, Asundexian, and EP-7041), which reversibly inhibit FXIa activity [83] (Table 3).

The principal factor XIa inhibitors investigated in clinical trials are:

- Asundexian: is a selective oral Factor XIa inhibitor administered once daily. It has a favorable pharmacokinetic profile, with minimal renal elimination and no significant dependence on hepatic cytochrome P450 metabolism. The Phase II trial, PACIFIC-AF [84], which enrolled patients with atrial fibrillation at increased risk of stroke, demonstrated a comparable antithrombotic effect of Asundexian to Apixaban but was associated with a significantly lower rate of bleeding with Asundexian. However, the following Phase III trials of the OCEANIC program, particularly the OCEANIC-AF [85], showed that treatment with Asundexian at a dose of 50 mg once daily resulted in a higher rate of stroke or systemic embolism compared to Apixaban, which led to the premature discontinuation of the trial. Despite this, Asundexian was associated with fewer major bleeding events over the course of the study.
- Milvexian: is another oral, highly selective Factor XIa inhibitor, taken once or twice daily. Like Asundexian, Milvexian is not renally cleared to a significant extent, and its metabolism does not rely heavily on hepatic enzymes. Initial Phase I studies established Milvexian’s safety, tolerability, and favorable pharmacokinetic profile [90]. The following Phase II study, AXIOMATIC-SSP trial, examined the safety and the efficacy of Milvexian versus placebo in patients with acute ischemic stroke or high-risk, showing no statistically significant reduction in the primary composite outcome of symptomatic ischemic stroke and covert brain infarction at 90 days [87]. The AXIOMATIC-TKR trial evaluated thromboprophylaxis after total knee replacement, showing that Milvexian significantly reduces the incidence of VTE in a dose-dependent manner, with bleeding rates similar to placebo and lower than those seen with enoxaparin [88]. The LIBREXIA Phase III program is ongoing with the aim of comparing Milvexian with standard care in the context of stroke prevention in nonvalvular AF (LIBREXIA-AF. NCT05757869), acute ischemic stroke, or high-risk TIA (LIBREXIA-STROKE. NCT05702034) and ACS (LIBREXIA-ACS. NCT05754957).
- Abelacimab: stands out among the Factor XIa inhibitors due to its monoclonal antibody structure, which allows it to bind to the inactive form of Factor XI and block its activation. It is administered parenterally (either intravenously or subcutaneously) and provides long-lasting anticoagulation with a monthly injection. In a first-in-human Phase 1 study, single subcutaneous administration of Abelacimab was demonstrated to be safe and well-tolerated [91]. The safety profile of Abelacimab was evaluated in a Phase II trial comparing intravenous doses to the standard of care (Enoxaparin) for the prevention of VTE [90]. In the Phase III AZALEA-TIMI 71 trial, which compared Abelacimab to Rivaroxaban in patients with atrial fibrillation, treatment with Abelacimab resulted in markedly lower levels of free factor XI and fewer bleeding events than treatment with Rivaroxaban [89].

According to these results, factor XIa inhibitors represent a major innovation in anticoagulation therapy: their selective mechanism of action, low renal clearance, and favorable pharmacology make them suitable for elderly patients with multiorgan functional decline even though dedicated studies are lacking. Abelacimab with its long-acting profile, could be especially compelling for frail or cognitively impaired elderly patients who struggle with daily oral therapy adherence. While not yet approved for clinical use, they may soon offer clinicians a valid option in the management of anticoagulation in the aging population.

## 11. Atherosclerotic Cardiovascular Disease and Concomitant Indication to Anticoagulant Therapy

In patients with ischemic heart disease undergoing percutaneous coronary revascularization with stent implantation, DAPT represents the most effective antithrombotic treatment [33,34]. However, it is not uncommon for these patients to also have an indication for anticoagulant therapy, either pre-existing or arising in the peri- or post-procedural phase. Among the possible reasons for anticoagulation, the most frequent is the coexistence of AF. It has been estimated that ischemic heart disease is present in about one-third of patients with AF, and conversely, 5–8% of patients undergoing coronary revascularization also have AF [1,92]. The coexistence of ASCVD and indication for anticoagulant therapy is burdened by poorer prognosis due to the higher risk of ischemic events, the increased number of pre-existing comorbidities, and the elevated risk of bleeding events. Consequently, a wide debate about the optimal therapy in this context is ongoing, especially regarding frail and older patients [93].

To address patients requiring both DAPT and OAT a triple antithrombotic therapy (TAT) (a combination of OAT and DAPT) has been historically proposed as the standard of care [94]. However, the increased likelihood of bleeding events with TAT often overcomes the potential benefits [95,96] (Table 4). This concern has led to the introduction of dual antithrombotic therapy (DAT), which combines OAT with a single antiplatelet agent [97].

**Figure 2:**
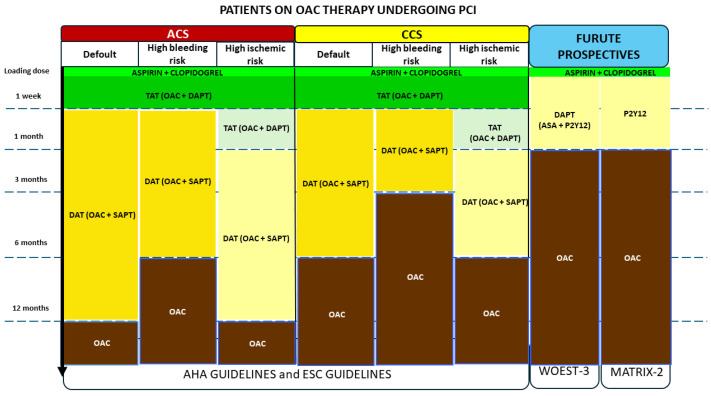
Atherosclerotic Cardiovascular Disease and Concomitant Indication to Anticoagulant Therapy. Current international guidelines recommend a brief course of TAT (one week), followed by the discontinuation of one antiplatelet agent, preferably aspirin, and continuation of DAT with a DOAC or VKA plus clopidogrel for up to 6 months in cases of CCS or up to 12 months in cases of ACS. After this period, patients are advised to continue OAT alone indefinitely. In patients at high bleeding risk, the duration of DAT can be reduced to 3 months in CCS and to 6 months in ACS. In patients at high ischemic risk, the duration of DAT may be prolonged; however, this option should be carefully evaluated in elderly patients due to their intrinsic concomitant risk of bleeding events. Ongoing RCTs are currently investigating therapeutic strategies aimed at further reducing bleeding risk without compromising ischemic protection, approaches that may be especially beneficial in the elderly population. ACS: Acute Coronary Syndrome, CCS: Chronic Coronary Syndrome, DAT: Dual Antithrombotic Therapy, DOAC: Direct Oral Anticoagulant, OAT: Oral Anticoagulant Therapy, RCT: Randomized Controlled Trial, TAT: Triple Antithrombotic Therapy, VKA: Vitamin K Antagonist.

Multiple randomized clinical trials have shown that DAT provides similar protection against thrombotic complications as TAT, but with a significantly lower risk of bleeding [98,99,100,101,102]. Consequently, current international guidelines recommend a brief course of TAT (ranging from 1 week to 1 month), followed by suspension of one antiplatelet agent, preferably Aspirin, continuing with a DAT represented by OAT and a P2Y12 for up to 6 months in cases of chronic coronary syndrome or up to 12 months in ACS. The P2Y12 inhibitor of choice should be Clopidogrel since the use of Prasugrel or Ticagrelor has been associated with a greater risk of major bleeding [103]. After this period, patients are advised to continue OAT alone indefinitely without antiplatelet therapy [110] (Figure 2).

The association between antiplatelet and anticoagulant therapy with DOACs or VKAs has been investigated in 4 large randomized and subsequent metanalyses, showing a lower bleeding risk, especially ICH, with DOACs [99,100,101,102,105].

Despite these results and recommendations from international guidelines, data from real-world registries indicate a different management, showing that TAT is yet frequently utilized for long periods [105,106,107,111], exposing patients to a higher risk of bleeding. This issue may become even more challenging in older patients due to their intrinsically higher hemorrhagic risk compared to younger populations.

A recent post hoc age-specific analysis of the PERSEO registry [110], aimed at comparing the clinical features, therapeutic strategies, and outcomes of individuals aged ≥80 and <80 years old treated with PCI and concomitant indication to anticoagulant therapy, showed that despite a higher baseline bleeding risk and the indications of current guidelines, more than 75% of patients older than 79 years were treated with a TAT strategy. DOACs were the anticoagulants of choice, but the 1-year follow-up bleeding rate (BARC 2–5) was very high (21%) among individuals ≥80 years old. Finally, the net adverse clinical events (NACE) rate was significantly higher in elderly patients due to a significantly higher rate of major adverse cardiac and cerebral events (MACCE) as well as major and clinically relevant bleeding compared to younger patients.

Two ongoing randomized clinical trials, WOEST-3 (What is the Optimal Antithrombotic Strategy in Patients with Atrial Fibrillation Undergoing PCI? NCT 04436978) [111] and MATRIX-2 (ClinicalTrials.gov Identifier: NCT05955365) are currently investigating new therapeutic strategies aimed at further reducing bleeding risk without compromising ischemic protection, which may be particularly beneficial for the elderly population. The WOEST-3 trial is a multicenter, open-label, randomized study investigating the safety and efficacy of one-month DAPT without oral anticoagulant therapy followed by DAT with oral anticoagulant and single antiplatelet therapy in patients with AF undergoing PCI. The MATRIX-2 trial is a multi-center, randomized trial on patients with AF requiring long-term oral anticoagulant therapy who have undergone successful PCI with stent implantation: patients will be randomized in a 1:1 ratio to the standard of care or a monotherapy with oral P2Y12 inhibitors immediately after PCI discontinuing Aspirin and DOAC. After 1 month, the P2Y12 inhibitor will be stopped, and treatment with a commercially available DOAC will be initiated for the duration of 11 months.

To sum up, antithrombotic therapy in patients undergoing PCI who also have an indication for OAT is associated with several clinical challenges due to the delicate balance between ischemic and bleeding risks. This is especially true in the elderly population, who are more vulnerable, highlighting the need for personalized treatment strategies. In this group of patients, a TAT strategy should be avoided due to the high bleeding risk. Future research efforts will address current gaps in evidence and support the development of patient-centered, evidence-based approaches for this high-risk population.

## 12. Conclusions

Antithrombotic therapy in elderly patients with cardiovascular disease poses significant challenges given the necessity to carefully balance ischemic protection against the increased risk of bleeding. Age-related factors such as frailty, comorbidities, and altered drug metabolism complicate treatment decisions, often in the absence of solid evidence, as older adults are underrepresented in clinical trials. In elderly patients, antithrombotic therapy should be less aggressive without compromising ischemic safety. When anticoagulant therapy is required, DAOCs should be preferred over vitamin K inhibitors due to their better safety profile. Recent advancements, such as new association strategies and emerging drugs, offer the promise of more targeted therapies with improved safety profiles.

## Figures and Tables

**Table 1:** Risk Factors for Thrombotic and Bleeding Events in the Elderly.

	Condition	Mechanism	Clinical Implications
**Thrombotic Risk Factors**	Increased platelet reactivity	↑ ADP-induced platelet aggregation	Enhanced thrombus formation
	Reduced antiplatelet activity	↓ PGI2; ↓ circulating PGI2	Higher platelet aggregation
	Endothelial dysfunction	↓ NO production	Reduced antiplatelet effect
	Increased intraplatelet ROS production	↑ NO degradation	Prothrombotic state
	Altered coagulation cascade	↑ Procoagulant factors	Hypercoagulability
	Chronic inflammatory state	Persistent activation platelets	Sustained prothrombotic state
	Reduced fibrinolytic activity	↑ PAI-1	Impaired clot dissolution
	**Condition**	**Mechanism**	**ClinicalImplications**
**Bleeding Risk Factors**	Vascular functional changes	↑ capillary fragility	Increased hemorrhagic risk
	Hepatic impairment	↓ drug metabolism and clearance	Accumulation of drugs
	Hypoalbuminemia	altered distribution	Enhanced drug activity
	Anemia	↓ platelet function	↓hemostatic response
	Previous GI bleeding	↑ mucosal vulnerability	Recurrence of bleeding
	Falls	↓ balance, vision, proprioception	Traumatic hemorrhage
	**Condition**	**Mechanism**	**ClinicalImplications**
**Combined thrombotic and ischemic risk factors**	Hypertension	↑ atherosclerosis and vasculopathy	Ischemic and hemorrhagic stroke
	Polypharmacy	Drug discontinuation/underuse	Therapeutic failure
	Renal impairment	Vascular disease + ↓ clearance of drugs	Ischemic and hemorrhagic events
	Previous vascular events	↑ thrombotic risk + fragile vasculature	Ischemic and hemorrhagic events

ADP: adenosine diphosphate, GI: gastrointestinal, PGI2: prostacyclin, ROS: reactive oxygen species. ↑ Increased; ↓ Decreased.

**Table 2:** Recommendations and dose adjustments of DOACs in elderly patients.

	Apixaban	Edoxaban	Rivaroxaban	Dabigatran
**Age ≥ 80 years**	2.5 mg BID if age ≥ 80 and ≤60 kg, or serum Cr ≥ 1.5 mg/dL	General caution advised	Caution in ≥75 years	Consider lower dose (110 mg BID) in ≥80 years
**Low body weight**	2.5 mg BID if ≤60 kg and age ≥ 80, or serum Cr ≥ 1.5 mg/dL	30 mg OD if ≤60 kg	General caution	Careful monitoring
**Renal impairment (CrCl 15–50 mL/min)**	2.5 mg BID if serum Cr ≥ 1.5 mg/dL and age ≥ 80, or ≤60 kg	30 mg OD if CrCl 15–50 mL/min	15 mg OD if CrCl 15–49 mL/min	Consider reduction to 110 mg BID
**Concomitant P-gp or CYP3A4 inhibitors**	Caution advised	30 mg OD	Caution advised	Caution advised
**Frailty**	Preferred agent	Suitable ts	Use with caution	Less preferred
**Cognitive impairment**	Well tolerated	Preferred for OD dose	Preferred for OD dose	Less preferred
**High GI bleeding risk/history of GI pathology**	Lower risk	Higher risk with high-dose	Higher risk	Higher risk vs. apixaban

BID: bis in die (twice a day), Cr: Creatinine, CrCl: Creatinine clearance, GI: Gastrointestinal, OD: one in die (once a day).

**Table 3:** New anticoagulants under development.

Main Author	Study	Year	Main Results
Piccini J.P. et al. (PACIFIC-AF) [84]	Randomized	2022	Asundexian reduced bleeding compared to Apixaban
Piccini J.P. et al. (OCEANIC-AF) [85]	Randomized	2025	Asundexian was associated with higher incidence of stroke or SE.
Sharma M. et al. (AXIOMATIC-SSP) [86]	Randomized	2024	Milvexian added to dual antiplatelet therapy did not increase the risk of major bleeding
Weitz J. et al. (AXIOMATIC-TKR) [87]	Randomized	2021	Milvexian reduces VTE rates with low bleeding risk compared to Enoxaparin
VerhammeP. et al. (ANT-005 TKA) [88]	Randomized	2021	Single intravenous dose of Abelacimab reduced bleeding compared to Enoxaparin.
Ruff C.T. et al. (AZALEA-TIMI 71) [89]	Randomized	2025	Abelacimab reduced bleeding events compared to Rivaroxaban
LIBREXIA-AF	Randomized	Ongoing	Milvexian vs. Apixaban in patients with Atrial Fibrillation

AF: Atrial Fibrillation, SE: Systemic Embolism, VTE: Venous Thromboembolism.

**Table 4:** Main studies on atherosclerotic cardiovascular disease and concomitant indication to anticoagulant therapy.

Main Author	Study	Year	Main Results
Lamberts M., et al. [95]	Registry	2012	High risk of bleeding with TAT after MI/PCI
Hess C.N. et al. [96]	Registry	2015	Higher rates of bleeding with TAT without difference in MI, death, or stroke vs. DAPT
Dewilde W.J.M. et al. M. et al. (WOEST) [98]	Randomized	2013	Less bleeding with clopidogrel without aspirin and no increase in the rate of thrombotic events
Gibson C.M. et al. (PIONEER-AF PCI) [99]	Randomized	2016	Rivaroxaban plus P2Y12 inhibitor reduced bleeding compared with VKA plus DAPT
Cannon C.P. et al. (REDUAL-PCI) [100]	Randomized	2017	Dabigatran + P2Y12 inhibitors reduces bleeding vs. TAT with warfarin
VranckxP. et al. (ENTRUST-AF PCI) [101]	Randomized	2019	Edoxaban + P2Y12 inhibitors was non inferior for bleeding vs. TAT with warfarin
Lopes L.D. et al. (AUGUSTUS) [102]	Randomized	2019	Apixaban + P2Y12 inhibitors reduces bleeding vs. TT with VKA
Andreou I., et al. [103]	Meta-analysis	2018	Ticagrelor in TAT increases bleeding complications compared to clopidogrel.
Gargiulo G., et al. [104]	Meta-analysis	2019	DAT reduces bleeding compared to TAT.
Chandrasekhar J. et al. (AVIATOR 2) [105]	Registry	2022	TAT was the most common strategy without differences in outcomes compared to DAT.
De Luca L. et al. (MATADOR-PCI) [106]	Registry	2021	TAT is yet frequently utilized for long periods
Sciahbasi A. et al. (PERSEO) [107]	Registry	2024	DAT reduces bleedings compared to TAT.
Minardi S. et al. (PERSEO-Elderly) [108]	Registry	2025	Higher risk of MACCE and bleeding in elderly patients on OAT post PCI
Verburg A. et al. (WOEST-3) [109]	Randomized	ongoing	1-month DAPT without oral anticoagulant therapy post PCI followed by DAT vs. SOC
Windecker S. et al. (MATRIX-2) (ClinicalTrials.gov Identifier: NCT05955365)	Randomized	ongoing	1-month monotherapy with oral P2Y12 inhibitors post PCI followed by DOAC vs. SOC

AF: Atrial Fibrillation; DAPT: Dual Antiplatelet Therapy; DAT: dual antithrombotic therapy; DOACs: Direct Oral Anticoagulants; MACCE: Major Adverse Cardiac and Cerebrovascular Events, MI: Myocardial Infarction, OAT: oral anticoagulant therapy; PCI: Percutaneous Coronary Intervention, SOC: Standard of Care; TAT: Triple Therapy, VKA: Vitamin K Antagonist.

## Data Availability

Data sharing is not applicable as no new data were created.
